# Supplementary material for: A UK survey of young people’s views on condom removal during sex
Source: PLoS One. 2024 Oct 23;19(10):e0298561. doi: 10.1371/journal.pone.0298561 (PMC11498692; doi:10.1371/journal.pone.0298561)
Supplement: S1 Fig — (DOCX) [file pone.0298561.s001.docx]

## **Supplementary Information**

## **
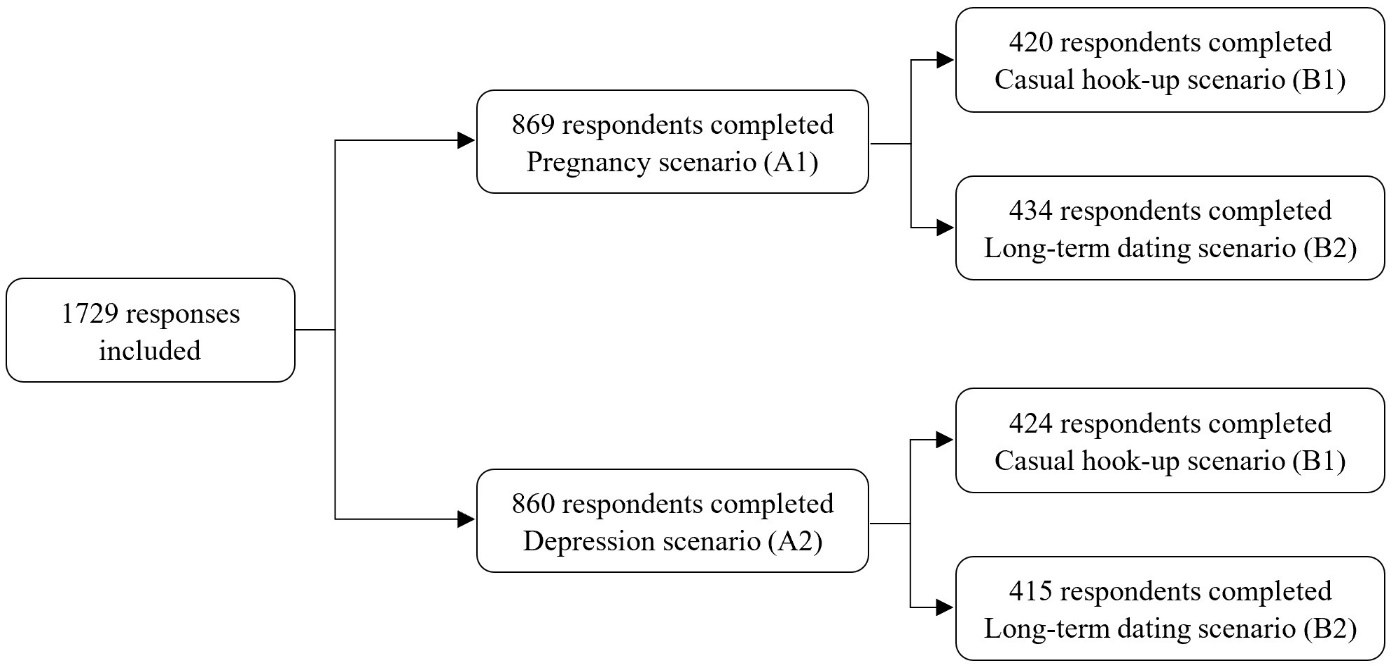
S1 Figure – Frequencies for allocated NCCR scenarios by outcome and relationship status**
