# Supplementary material for: A UK survey of young people’s views on condom removal during sex
Source: PLoS One. 2024 Oct 23;19(10):e0298561. doi: 10.1371/journal.pone.0298561 (PMC11498692; doi:10.1371/journal.pone.0298561)
Supplement: S1 Table — (DOCX) [file pone.0298561.s002.docx]

## **Supplementary Information**

## **S1 Table – Factors associated with agreeing that prison was an appropriate penalty for Sam in the Relationship Status scenario: logistic regression model including interaction term**

| **Variable** | **Adjusted** | | |
| --- | --- | --- | --- |
|  | **OR (95% CI)** | **P-value** | |
| **Relationship status** | | | |
| **Casual hook-up** | **1.0** | **0.05** | |
| **Long-term dating** | **0.76 (0.58 – 1.00)** |  |  |
| **Outcome** | | | |
| **Pregnancy** | **1.0** | **0.02** |  |
| **Depression** | **0.72 (0.55 – 0.95)** |  |  |
| **Interaction term** |  |  |  |
| **Depression/ Long-term dating** | **1.00 (0.67 – 1.47)** | **0.99** |  |
| **Sex** | | | |
| **Male** | **1.0** | **<0.001** | |
| **Female** | **1.62 (1.28 – 2.06)** |  |  |
| **Sexual orientation** | | | |
| **Heterosexual** | **1.0** | **0.005** | |
| **Gay or lesbian** | **1.05 (0.71 – 1.53)** |  |  |
| **Bisexual** | **1.46 (1.17 – 1.81)** |  |  |
| **Other sexual orientation** | **1.43 (0.96 – 2.13)** |  |  |
