# Supplementary material for: A UK survey of young people’s views on condom removal during sex
Source: PLoS One. 2024 Oct 23;19(10):e0298561. doi: 10.1371/journal.pone.0298561 (PMC11498692; doi:10.1371/journal.pone.0298561)
Supplement: S2 Table — (DOCX) [file pone.0298561.s003.docx]

## **Supplementary Information**

## **S2 Table –** **Chi-square test results exploring associations between sociodemographic factors and scenario allocation**

| **S2A Table – Frequencies and chi-square test results exploring associations between Outcome scenario allocation and socio-demographic characteristics** | | | |
| --- | --- | --- | --- |
| **Socio-demographic characteristic** | **Pregnancy** n (%) | **Depression** n (%) | **P-value** |
| **Sex** |  |  |  |
| Male | 188 (22.4%) | 214 (25.8%) | 0.10 |
| Female | 652 (77.6%) | 614 (74.2%) |  |
| **Gender identity** |  |  |  |
| Cisgender | 753 (89.5%) | 744 (89.9%) | 0.83 |
| Other gender identity | 88 (10.5%) | 84 (10.1%) |  |
| **Sexual** **orientation** |  |  |  |
| Straight or heterosexual | 359 (42.7%) | 396 (47.8%) | 0.20 |
| Gay or lesbian | 71 (8.4%) | 60 (7.2%) |  |
| Bisexual | 347 (41.3%) | 317 (38.3%) |  |
| Other sexual orientation | 64 (7.6%) | 55 (6.6%) |  |
| **Ethnicity** |  |  |  |
| White | 713 (85.0%) | 703 (84.9%) | 0.96 |
| Non-White | 126 (15.0%) | 125 (15.1%) |  |
| **Belonging** **to** **a** **religion** |  |  |  |
| Yes | 140 (16.7%) | 146 (17.7%) | 0.60 |
| No | 699 (83.3%) | 681 (82.3%) |  |
| **Born** **in** **the** **UK** |  |  |  |
| Yes | 690 (82.8%) | 685 (83.2%) | 0.83 |
| No | 143 (17.2%) | 138 (16.8%) |  |

| **S2B Table – Frequencies and chi-square test results exploring associations between Relationship status scenario and socio-demographic characteristics** | | | |
| --- | --- | --- | --- |
| **Socio-demographic characteristic** | **Casual hook-up** n (%) | **Long-term dating** n (%) | **P-value** |
| **Sex** |  |  |  |
| Male | 209 (25.2%) | 193 (23.0%) | 0.28 |
| Female | 619 (74.8%) | 647 (77.0%) |  |
| **Gender identity** |  |  |  |
| Cisgender | 735 (88.8%) | 762 (90.6%) | 0.22 |
| Other gender identity | 93 (11.2%) | 79 (9.4%) |  |
| **Sexual orientation** |  |  |  |
| Straight or heterosexual | 347 (41.9%) | 408 (48.5%) | 0.05 |
| Gay or lesbian | 73 (8.8%) | 58 (6.9%) |  |
| Bisexual | 345 (41.7%) | 319 (37.9%) |  |
| Other sexual orientation | 63 (7.6%) | 56 (6.7%) |  |
| **Ethnicity** |  |  |  |
| White | 707 (85.5%) | 709 (84.4%) | 0.54 |
| Non-White | 120 (14.5%) | 131 (15.6%) |  |
| **Belonging to a religion** |  |  |  |
| Yes | 130 (15.7%) | 156 (18.6%) | 0.13 |
| No | 696 (84.3%) | 684 (81.4%) |  |
| **Born in the UK** |  |  |  |
| Yes | 690 (84.1%) | 685 (81.9%) | 0.23 |
| No | 130 (15.9%) | 151 (18.1%) |  |
