# Supplementary material for: A UK survey of young people’s views on condom removal during sex
Source: PLoS One. 2024 Oct 23;19(10):e0298561. doi: 10.1371/journal.pone.0298561 (PMC11498692; doi:10.1371/journal.pone.0298561)
Supplement: Attachment — Submitted filename: ResponsetoReviewer3.docx [file pone.0298561.s005.docx]

**Response to Reviewer and Editor** (*Manuscript: A UK survey of young people’s views on condom removal during sex)*

*Reviewer comment is in bold, our response is in normal text.*

**Reviewer #1: Dear authors,

Thank you for addressing all my comments. However, there are still issues with the Introduction as it feels to be too short and not in-depth enough. I'd like to see a short description of the methods used in the research you have chosen to include in your Introduction. At the moment it is hard to understand how the previous research has been conducted, i.e. qual or quant etc. There is no need to present the info as a systematic review (because this is not what you did), but you need to present the info in a more systematic way.

Other than that, I am satisfied with the way you have addressed my comments. Thank you!**

We would like to thank the reviewer for their careful reading of our paper.

We have added additional detail of the methodologies of the studies cited in the introduction in two paragraphs of the revised paper. We also note that our statements are referenced, and it is possible for readers to find and read the original research that is being cited if they wish to know more details about the research.

In terms of style and length, our introduction is deliberately in keeping with other recent sexual health-related *PloS ONE* papers, e.g.:

<https://journals.plos.org/plosone/article?id=10.1371/journal.pone.0259182>

<https://journals.plos.org/plosone/article?id=10.1371/journal.pone.0282187>

<https://journals.plos.org/plosone/article?id=10.1371/journal.pone.0189607>
